# Regioselectivity of 1,3-dipolar cycloadditions and antimicrobial activity of isoxazoline, pyrrolo[3,4-*d*]isoxazole-4,6-diones, pyrazolo[3,4-*d*]pyridazines and pyrazolo[1,5-*a*]pyrimidines

**DOI:** 10.1186/s13065-016-0163-2

**Published:** 2016-04-01

**Authors:** Yasser Hassan Zaki, Abdelwahed Rashad Sayed, Shaaban A. Elroby

**Affiliations:** Department of Chemistry , Faculty of Science, Beni-Suef University, Beni-Suef, 62514 Egypt; Department of Chemistry, Faculty of Science, KFU, Hofuf, Saudi Arabia; Department of Chemistry, Faculty of Science, King Abdulaziz University, Jeddah, 21589 Saudi Arabia

**Keywords:** Isoxazoline, Regioselectivity, 1,3-Dipolar cycloadditions, Density functional theory, Pyrrolo[3,4-*d*]isoxazole, Pyrazoles, Pyrazolo[3,4-*d*]pyridazines, Pyrazolo[1,5-*a*]pyrimidine

## Abstract

**Background:**

Isoxazoles exhibit interesting biological activities, and the 1,3-dipolar cycloaddition(13DC) reactions play an important role in both mechanistic and synthetic organic chemistry. Pyrazoles and annulated pyrazoles exhibit some diverse biological activities. They are used as antipyretic, analgesic drugs, tranquilizing, and herbicidal agents. Pyrazoles are also used extensively as useful synthons in organic synthesis. Pyrazolo[3,4-*d*]pyridazines showed good antimicrobial, anti-inflammatory and analgesic activities. Several oximes are found to be hyperglycemic, anti-neoplastic, anti-inflammatory, anti-leishmanial and VEGFR-2 kinase inhibitors.

**Results:**

The present work describes an efficient synthesis protocol and molecular orbital calculations of isoxazoline and pyrrolo[3,4-*d*]isoxazole-4,6-dione derivatives from the reaction of hydroximoyl chloride with acrylonitrile, acrylamide, and *N*-arylmalemides. In addition, pyrazoles and pyrazolo[3,4-*d*]pyridazines are obtained via the reaction of 3-(dimethylamino)-1-(2,4-dimethyl-1,3-thiazol-5-yl)prop-2-ene-1-one with hydrazonoyl halides. Pyrazolo[1,5-*a*]pyrimidines were derived from condensation of either Sodium Salt of 3-Hydroxy-1-(2,4-dimethylthiazol-5-yl)prop-2-en-1-one (**10**) or 3-(dimethylamino)-1-(2,4-dimethyl)(1,3-thiazol-5-yl)prop-2-en-1-one (**11**) with aiminopyrozoles. A comparative study of the biological activity of the synthesized compounds with ampicillin and tetracycline is compiled in Table 3. Generally, all synthesized compounds showed an adequate inhibitory efficiency of growth of gram-positive and gram-negative bacteria. Structures of the newly synthesized compounds were elucidated by elemental analysis, spectral data and a computational study.

**Conclusions:**

In summary, new and efficient synthetic routes of isoxazoline, pyrrolo[3,4-*d*]isoxazole-4,6-dione derivatives, pyrazoles, pyrazolo[3,4-*d*]pyridazines and pyrazolo[1,5-*a*]pyrimidines have been achieved and the biological activity has been investigated.

**Graphical abstract:**
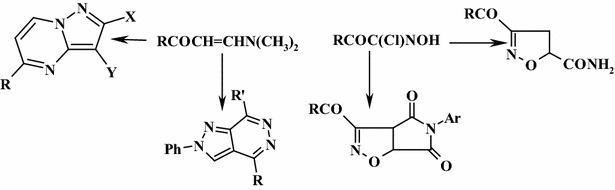
New and efficient synthetic routes of isoxazoline, pyrrolo[3,4-*d*]isoxazole-4,6-dione derivatives, pyrazolo[3,4-*d*]pyridazines and pyrazolo [1,5-*a*] pyrimidines

## Background

Isoxazoles exhibit interesting biological activities [1, 2], and oxazoles are widely recognized for their therapeutic purposes, especially as tranquillizing agents and CNS regulates. They are known to have bacteriostatic, bactericidal and fungicidal activities [3]. The 1,3-dipolar cycloaddition(13DC) reactions play an important role in both mechanistic and synthetic organic chemistry. Pyrazoles and annulated pyrazoles exhibit some diverse biological activities. They are used as antipyretic [4], analgesic drugs [5–7], tranquilizing [8] and herbicidal [9] agents. Pyrazoles are also used extensively as useful synthons in organic synthesis [10–17]. Recently, the synthesis of biologically active compounds based on pyrazolo[3,4-*d*]pyridazines systems are of outstanding importance for medicinal and biological utilities [18, 19], Generally, pyrazolo[3,4-*d*]pyridazines showed good antimicrobial, anti-inflammatory and analgesic activities [20]. Herein, we report a facile synthesis procedure for some new derivatives of the newly developed isoxazoline, pyrrolo[3,4-*d*]isoxazole-4,6-dione derivatives, pyrazoles, pyrazolo[3,4-*d*]pyridazines and pyrazolo[1,5-*a*] pyrimidines.

## Results and discussion

### Chemistry

**Scheme 1:**
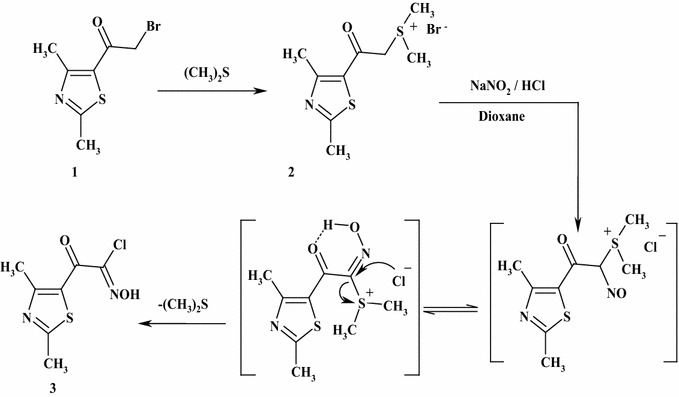
Synthesis of 2-chloro-2-(hydroximino)-1-(2,4-dimethylthiazol-5-yl)ethanone (**3**)

The reaction of 1-(2,4-dimethyl(1,3-thiazol-5-yl))-2-bromoethan-1-one (**1**) [21] with dimethylsulfide in refluxing ethanol has afforded 1-(2,4-dimethylthiazol-5-yl)-2-oxodimethylsulfonium bromide (**2**) [21], furthermore, the nitrosation of (**2**) in dioxane-water solution acidified with hydrochloric acid gave 2-chloro-2-(hydroximino)-1-(2,4-dimethyl-1,3-thiazol-5-yl)ethanone (**3**). The chemical structure of (**3**) was confirmed based on elemental analysis, spectral data, and chemical transformations. The ^1^H NMR spectrum showed signals at *δ* = 2.47 (s, 3H, CH_3_), 2.71 (s, 3H, CH_3_) and 13.18 (s, 1H, NOH). The IR spectrum revealed absorption bands at 3370 cm^−1^ (OH) and 1655 cm^−1^ (CO conjugated) (Scheme 1).

**Scheme 2:**
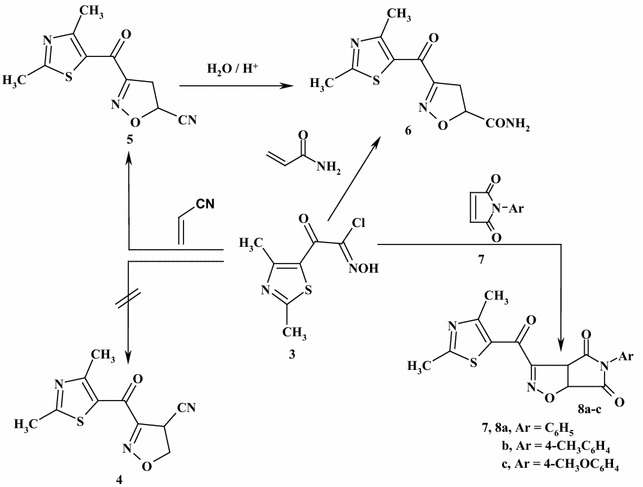
Synthesis of isoxazoline, pyrrolo[3,4-*d*]isoxazole **5** and (**8a**–**c**)

Moreover, the treatment of 2-chloro-2-(hydroximino)-1-(2,4-dimethylthiazol-5-yl)ethanone (**3**) with acrylonitrile in boiling toluene afforded an insoluble product, according to *TLC*, of which structures (**4**) and (**5**) seemed to be possible (Scheme 2). The ^1^H NMR spectrum of the product showed signals at *δ* = 2.57 (s, 3H, CH_3_) 2.80, (s, 3H, CH_3_), 2.90–2.94 (d, 2H, CH_2_, *J* = 10 Hz, isoxazoline C-4) and 3.85 (t, 1H, *J* = 10 Hz, isoxazoline C-5). Its IR spectrum revealed bands at 1665 cm^−1^ (CO). However, no absorption bands appeared at 2200 cm^−1^ corresponding to the CN group in support of the 5-cyano structure [22]. The product was readily hydrolyzed by dilute sulfuric acid to give the corresponding 3-[(2,4-dimethyl-1,3-thiazol-5-yl)carbonyl]-4,5-dihydroisoxazole-5-carboxamide (**6**) (IR spectral bands at 3350, 3170 cm^−1^ (NH_2_) and 1680 cm^−1^ (CO)). In addition, refluxing of 2-chloro-2-(hydroximino)-1-(2,4-dimethylthiazol-5-yl)ethanone (**3**) with acrylamide in a boiling toluene furnished an identical product with compound (**6**) in all aspects (m.p., mixed m.p., spectra). Hence, the proposed structure (**4**) was excluded and the product was assigned to a formulated structure of 3-[(2,4-dimethyl-1,3-thiazol-5-yl)carbonyl]-4,5-dihydro-isoxazole-5-carbonitrile (**5**). Also, the compound (**3**) was reacted with the appropriate *N*-arylmalemides (**7a**–**c**) in boiling toluene and produced 3-[(2,4-dimethyl-1,3-thiazol-5-yl)carbonyl]-5-substituted-3(a*H)*-pyrrolo[3,4-*d*]isoxazole-4,6 (5*H*,6a*H*)dione (**8a**-**c**), respectively (Scheme 2).

The structures of compounds (**8**) were confirmed by elemental analysis and spectral data. The IR spectra of (**8a**–**c**) revealed bands at 1730 and 1637 cm^−1^ assigned for CO and -CO-NAr-CO- groups [23]. The ^1^H NMR spectrum of (**8a**) showed signals at *δ* = 2.66 (s, 3H, CH_3_), 2.92 (s, 3H, CH_3_), 5.23–5.24 (d, 1H, *J* = 7.4 Hz, isoxazoline C-4), 5.81–5.88 (d, 1H, *J* = 7.4 Hz, isoxazoline C-5), and 7.22–7.33 (m, 5H, ArH’s).

## Quantum chemical calculations

### Computational methods

All calculations have been carried out using the Gaussian 09 suite of programs [24]. The geometries of the reactants, transition states and products have been fully optimized at the DFT/B3LYP/6-311 ++G** level of the theory [25–28]. Frequency calculations were performed at the same level of the theory in order to characterize the stationary points and to evaluate the zero-point energy (ZPE), free energies (G) and enthalpies (H) at 298.15 K. TSs had only one imaginary frequency.

**Scheme 3:**
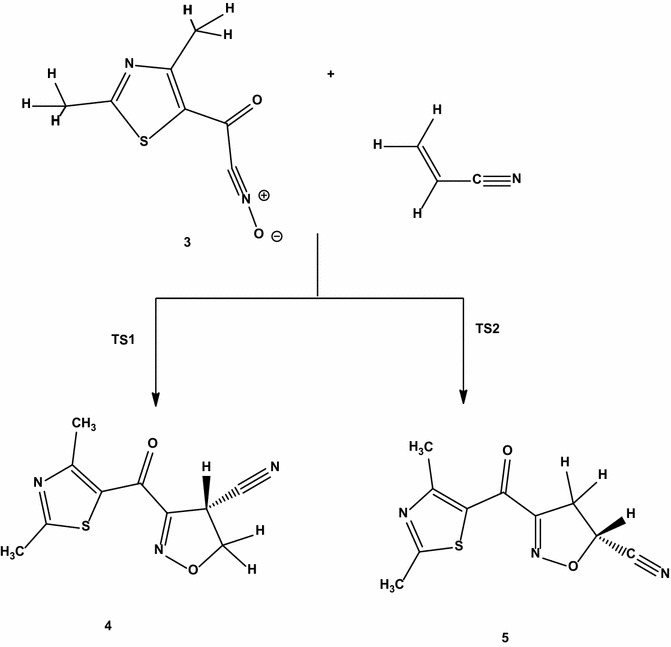
Regioisomeric pathways for the studied cycloaddition reaction

**Fig. 1:**
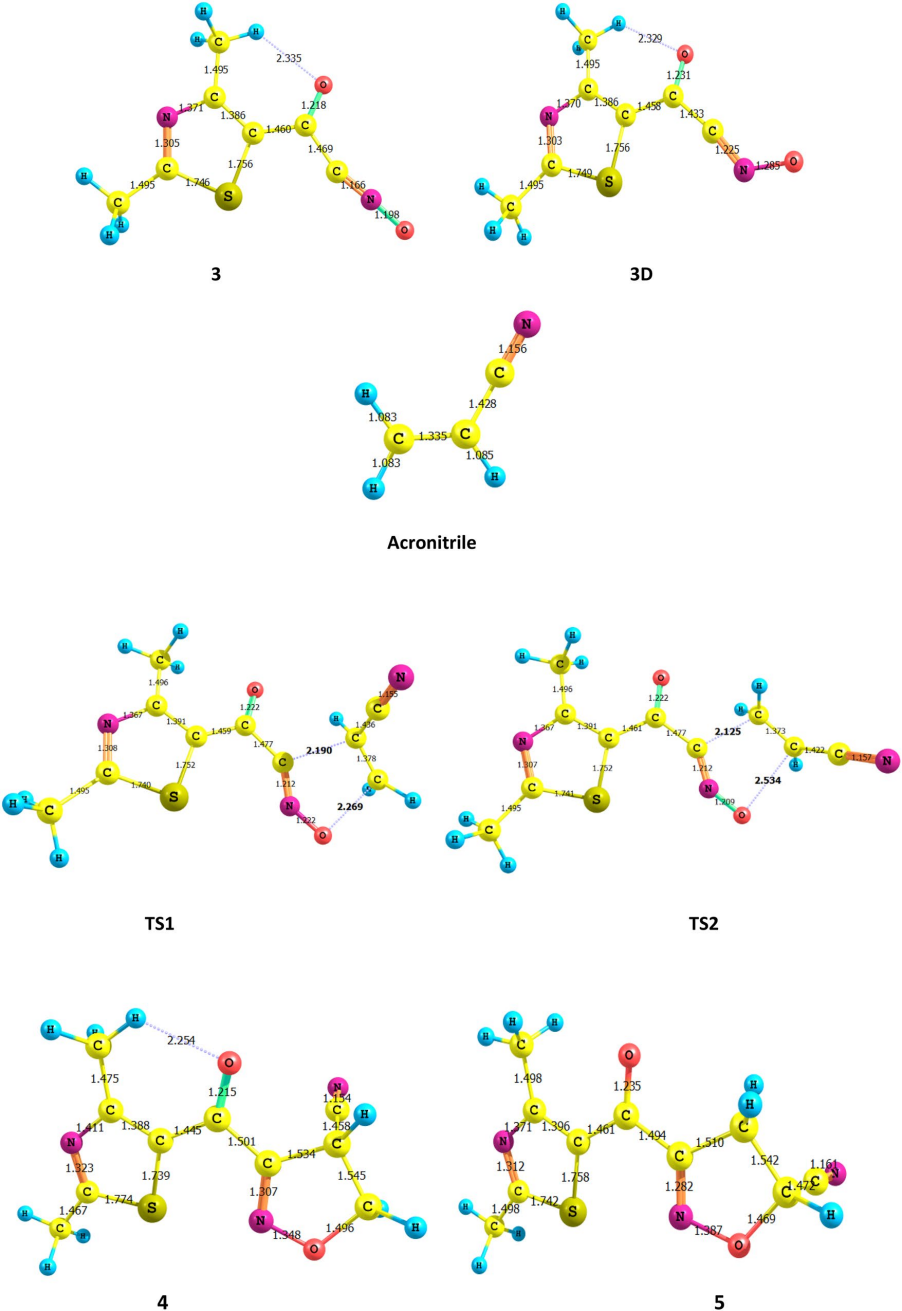
Optimized geometries obtained by B3LYP/6-311 ++G** of all spices in the studied 13DC reaction. The bond lengths are given in angstroms

The interaction between acrylonitrile and 2,4-dimethylthiazol (13DC) can give two isomeric structure **5** (head-to-head) or **4** (head-to-tail) as shown in Scheme 3. There are some theoretical studies of the 1,3-dipolar cycloadditions of carbon materials [29–31]. Density functional theory (DFT) is employed to investigate the **13DC** reaction. We report a computational study of regioselectivity of 2,4-dimethylthiazol (**3**) cycloadditions to acrylonitrile dipolarophiles. Our main objective in obtaining these results is to calculate the energy barrier for the 13DC reaction. B3LYP method confirms that the **5** geometry is preferred by 3.789 kJ mole^−1^, see Table 1. Our results are in complete agreement with experimental which indicated that the **5** conformer is the product from the above reaction. The calculated geometries of the stationary points corresponding to this **13DC** reaction (reactants, transition states, and products) are presented in Fig. 1. The total and relative energies are shown in Tables 1 and 2. The TSs structures from B3LYP calculations for **13DC** are very similar with minor changes in the bond distances and energies, see Tables 1 and 2. Four bond distances are important in 13 DC reaction. Two existing double bonds elongate (C=C and C=N) and two new bonds form (C–C and C–O) during this cycloaddition reaction. The C–C bond lengths of the acrylonitrile are only ~0.02 longer in the transition states than in the reactant. Similarly, the C–N and N–O bond lengths of structure **3** are only changed by 0.013 Å longer in the transition states than in the reactant. It is clear to note that, each transition state involves significant bending of structure **3** angle from its planar ground-state geometry to a product-like bending angle. The distorted structure for **3** structure with bending angle is named **3D** as presented in Fig. 1. The bending angle changes from the ground state to the transition state range from 180° to ~120°. The distortion energy for structure **3** is 199.247 kJ mole^−1^. The corresponding activation barrier, enthalpy, free energy and reaction energies are given in Table 2. As mentioned before, the studied **13DC** reaction favors structure **5** product with the lower activation energy (72.328 kJ mole^−1^) and high negative values of enthalpy and free energy.

**Table 1:** Zero point energy (ZPE), electronic energy (E), enthalpy (H) and free energies (G), total energy (E + ZPE) computed at 298 K of the stationary points involved in the studied 13DC reaction using B3LYP/6-311 ++G** level of theory

Structure	ZPE	E	H	G	Et
au					
**2**	0.05058	−170.88289	−170.82720	−170.85818	−170.83231
**3**	0.12172	−928.57717	−928.44370	−928.49390	−928.45545
**3D**	0.11855	−928.49839	−928.36910	−928.41635	−928.37984
**TS1**	0.17372	−1099.43404	−1099.24270	−1099.30732	−1099.26032
**TS2**	0.17355	−1099.43868	−1099.24740	−1099.31274	−1099.26513
**4**	0.17903	−1099.50842	−1099.30020	−1099.37243	−1099.32939
**5**	0.17858	−1099.50940	−1099.31520	−1099.37487	−1099.33082

**Table 2:** Enthalpies (∆H) and free energies (∆G), barrier energies $\left( {{\text{E}}^{\text{f}}_{\text{a}} ,{\text{E}}^{\text{b}}_{\text{a}} } \right)$, relative energies (∆E) computed at 298 K of the stationary points involved in the studied 13DC reaction using B3LYP/6-311 ++G** level of theory

Product	∆H	∆G	${\text{E}}^{\text{f}}_{\text{a}}$	${\text{E}}^{\text{b}}_{\text{a}}$	∆E
kJ/mole					
**4**	−273.437	−59.468	−72.328	−182.020	3.789
**5**	−274.012	−60.043	−65.549	−173.141	0

**Fig. 2:**
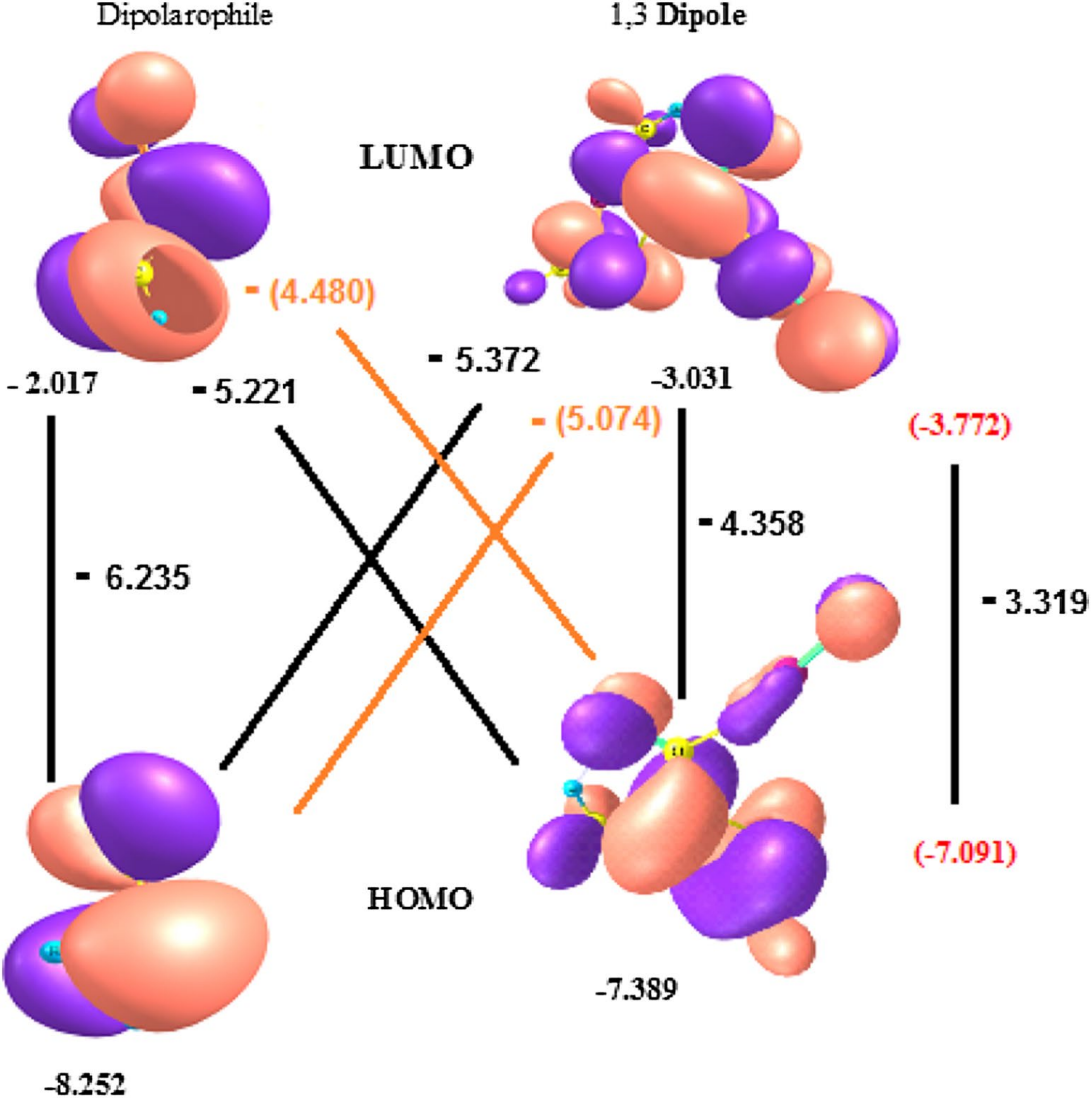
FMO interactions in 13DC and the HOMO, LUMO, gap energy (HOMO–LUMO) and interaction separation energies (HOMO–LUMO) (eV) are calculated at B3LYP/6-311 ++G**. Values given in* red color* are for distortion structure 3D

The frontier molecular orbital (FMO) obtained by B3LYP/6-311 ++G** level of the theory of the studied molecules are plotted in Fig. 2. The energies and shape of the FMO (HOMO and LUMO) for both 1,3-dipole **3** and dipolarophile determine the chemical reactivity in cycloaddition reactions. Hence, the interaction between the FMO is important to rationalize of the cycloaddition processes. The computations demonstrate that the distortion structure 3 to give 3D structure decrease HOMO–LUMO separation energy, which is capable to react with acrylonitrile. When the separation energy between the interaction orbital small, the better they interact. Comparing the energies of the FMO, HOMO–LUMO of the dipolarophile and **3**, we can suggest that 13DC reaction as HOMO for **3** controlled. The interaction of the dipole HOMO with the dipolarophile LUMO is greater, due to small separation interaction energy between them. The computations show that the structure **3** has a small HOMO–LUMO gap energy (4.358 eV) compared to 3D structure (3.319 eV). As shown in Fig. 2, the interaction energy separation between HOMO of **3D** (1,3 dipole) and LUMO of dipolarophile is 4.480 eV compared to the value of HOMO for **3** and LUMO for dipolarophile which it is 5.074 eV. It is shown from the calculations that the reaction of **3 structure** (1,3 dipolar) with acrylonitrile (dipolarophiles) is occurred during charge transfer from HOMO of structure **3** to LUMO of acrylonitrile. With respect to the shape of HOMO and LUMO, only if the interacting lobes are in phase the reaction is thermally feasible. The lobes of HOMO for **3D** and LUMO of dipolarophile are in the same phase, which it is thermally feasible.

**Scheme 4:**
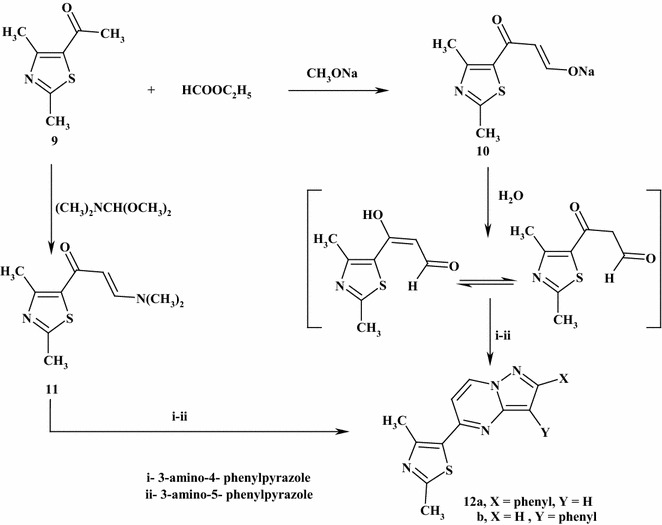
Synthesis of pyrazolo[1,5-*a*]pyrimidines (**12a**,**b**)

Besides, the compound 1-(2,4-dimethyl-1,3-thiazol-5-yl)ethanone (**9**) was reacted with ethyl formate in dried ether containing sodium methoxide and afforded the sodium salt of 3-hydroxy-1-(2,4-dimethylthiazol-5-yl)prop-2-en-1-one (**10**). The structure of compound (**10**) was elucidated by its chemical transformations. Furthermore, treatment of compound (**10**) with 3-amino-4-phenyl-1*H*-pyrazole or 3-amino-5-phenyl-1*H*-pyrazole in glacial acetic acid containing piperidenum acetate afforded compounds 5-(2,4-dimethyl-1,3-thiazol-5-yl)-3(or 4)-phenylpyrazolo[1,5-*a*]pyrimidines (**12a**,**b**), respectively (Scheme 4). The structures of compounds (**12a**,**b**) were elucidated by elemental analysis, spectral data, and an alternate synthetic route. The ^1^H NMR spectrum of (**12a**) showed signals at *δ* = 2.59 (s, 3H, CH_3_), 2.83 (s, 3H, CH_3_), 6.53 (s, 1H, pyrazole H-4), 7.13 (d, 1H, *J* = 4 Hz, pyrimidine H-5), 7.54–7.92 (m, 5H, ArH’s) and 8.74 (d, 1H, 8 Hz, pyrimidine H-6). On the other hand, refluxing of 5-acetyl-2,4-dimethylthiazole (**9**) with dimethylformamide-dimethylacetal in boiling dry xylene gave the compound 3-(dimethylamino)-1-(2,4-dimethyl-1,3-thiazol-5-yl))prop-2-ene-1-one (**11**) in a good yield. Chemical elucidation of compound (**11**) was confirmed by elemental analysis, spectral data, and chemical transformation. The ^1^H NMR spectrum showed signals at *δ* = 2.49 (s, 3H, CH_3_), 2.78 (s, 3H, CH_3_), 2.98 (s, 3H, CH_3_), 3.15 (s, 3H, CH_3_), 5.49–5.54 (d, 1H, *J* = 12 Hz, CH=CH–N) and 6.90–7.28 (d, 1H, *J* = 12 Hz, CH=CH–N). Further reaction of the compound (**11**) with 3-amino-4-phenylpyrazole or 3-amino-5-phenyl-1*H*-pyrazole in a mixture of acetic acid and ammonium acetate gave identical products in all aspects (m.p., mixed m.p., spectra) to (**12a**,**b**) (Scheme 4).

**Scheme 5:**
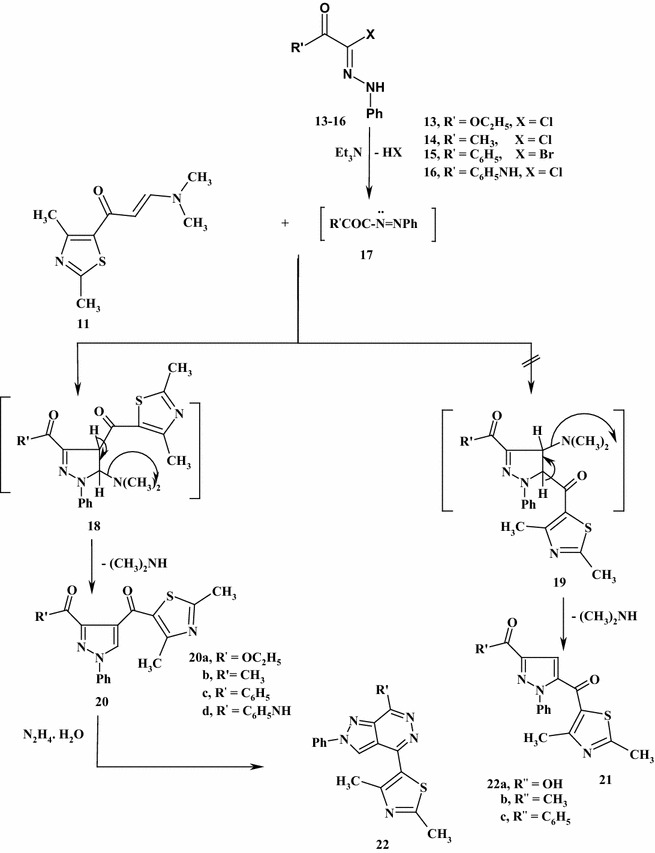
Synthesis of pyrazolo[3,4-*d*]pyridazines (**22a**–**c**)

Finally the treatment of *C*-ethoxycarbonyl-*N*-phenylhydrazonoyl chloride [32–35] (**13**) with compound (**11**) in refluxing toluene containing triethylamine catalyst yielded a new isolable product, which formulated as either ethyl 3-[(2,4-dimethyl-1,3-thiazol-5-yl)carbonyl]-1-phenyl-pyrazole-4-carboxylate (**20a**) or ethyl 3-[(2,4-dimethyl-1,3-thiazol-5-yl)carbonyl]-1-phenyl-pyrazole-5-carboxylate (**21a**) (Scheme 5). The structures of compounds (**20**) were elucidated by their spectral, elemental analysis and chemical transformation. The ^1^H NMR spectrum of compound (**20a**) showed a characteristic signals at *δ* = 1.22 (t, 3H, CH_3_, *J* = 7 Hz), 2.44 (s, 3H, CH_3_), 2.69 (s, 3H, CH_3_), 4.33 (q, 2H, CH_2_, *J* = 7 Hz), 7.44–7.89 (m, 5H, ArH’s) and 8.19 (s, 1H, pyrazole C-5). Formation of compounds (**20**) can be verified via chemical reaction of nitrilum imide (**17**), formed in situ from hydrazonoyl halides and triethylamine, with compound (**11**) which afforded the cycloadduct intermediate (**18**) or (**19**). After elimination of dimethylamine, the pyrazoles were obtained as final products (**20**) or (**21**). Similarly, the appropriate hydrazonoyl halides (**14**–**16**) reacted with a compound (**11**) to afford the corresponding pyrazoles (**20b**–**d**).

Boiling of the appropriate pyrazoles (**20a**–**d**) with hydrazine hydrate in ethanol yielded Pyrazolo[3,4-*d*]pyridazines (**22a**–**c**) (Scheme 5). The chemical structures of compounds (**22a**–**c**) were elucidated via elemental analysis, spectral data and alternative synthesis. The ^1^H NMR spectrum of compound (**22b**) depicted signals at *δ* = 2.46 (s, 3H, CH_3_), 2.77 (s, 3H, CH_3_), 3.01 (s, 3H, CH_3_), 7.32–8.11 (m, 5H, ArH’s), 8.76 (s, 1H, pyrazole C-5). Alternatively, a new route for the synthesis of the compound (**22a**), the compound (**20d**) was refluxed with hydrazine hydrate in ethanol to give an identical product in all aspects (m.p., mixed m.p., and spectra) with compound (**22a**).

## Conclusions

In summary, new and efficient synthetic routes of isoxazoline, pyrrolo[3,4-*d*]isoxazole-4,6-dione derivatives, pyrazoles, pyrazolo[3,4-*d*]pyridazines and pyrazolo [1,5-*a*] pyrimidines have been achieved, and computational investigations are in complete agreement with experimental. Moreover, the selected newly synthesized products were evaluated for their antimicrobial activity against gram positive and gram negative bacteria as well as some fungal-plants. The results revealed all synthesized compounds showed an adequate inhabitory efficiency of growth of gram positive and gram negative bacteria.

## Experimental section

### General methods

All melting points were determined on an electrothermal apparatus and are uncorrected. IR spectra were recorded (KBr discs) on a Shimadzu FT-IR 8201 PC spectrophotometer. ^1^H NMR and ^13^C NMR spectra were recorded in CDCl_3_ and (CD_3_)_2_SO solutions on a Varian Gemini 300 MHz spectrometer, and chemical shifts are expressed as δ using TMS as an internal reference. Mass spectra were recorded on a GC–MS QP1000. Elemental analyses were carried out at the Microanalytical Center of Cairo University. The hydrazonoyl halides [32–35] and hydroximoyl chloride [36] were prepared as previously described.

### Synthesis of 2-chloro-2-(hydroximino)-1-(2,4-dimethyl)thiazol-5-yl)ethanone (**3**)

Hydrochloric acid (12 M, 100 ml) was added while stirring to a mixture of 1-(2,4-dimethylthiazol-5-yl)-2-oxodimethyl-sulfonium bromide (**2**) (11.8 g, 0.04 mol), sodium nitrite (3.5 g, 0.05 mol) in 1,4-dioxane (50 ml) and water (50 ml) at 25 °C. Stirring was continued for 3 h to produce a pale yellow solid, which was separated by filtration and recrystallized from ethanol to give (**3**). Yellow solid; Yield (62 %); m.p. 142 °C. IR (KBr) ν_max_: 3370 (OH), 3055, 2966 (CH), 1655 (CO conjugated) cm^−1^; ^1^H NMR (DMSO-*d*_6_): 2.47 (s, 3H, CH_3_), 2.71 (s, 3H, CH_3_) and 13.18 (s, 1H, NOH); MS *m/z* (%): 218 (M^+^, 100). Anal. Calcd for C_7_H_7_ClN_2_O_2_S (218.66): C, 38.45; H, 3.23; N, 12.81; S, 14.66; Found: C, 38.43; H, 3.22; N, 12.79; S, 14.64 %.

### Synthesis of 3-[(2,4-dimethyl-1,3-thiazol-5-yl)carbonyl]-4,5-dihydroisoxazole-5-carbonitrile (**5**), 3-[(2,4-dmethy-1,3-thiazol-5-yl)carbonyl]-4,5-dihydroisoxazole-5-carboxamide (**6**) and 3-[(2,4-dimethyl-1,3-thiazol-5-yl)carbonyl]-5-substituted 3aH-pyrrolo[3,4-d]isoxazole-4,6(5H,6aH)-dione (**8a**-**c**)

#### General method

Equimolar amounts of the appropriate (**3**), acrylonitrile, acrylamide or the appropriate *N*-arylmalemides (**7a**–**c**) (0.005 mol each) in toluene (30 ml) were heated under reflux for 18 h. The solvent was evaporated under vacuum and the residual oil was triturated with petroleum ether (40–60 °C). The solid products were collected and recrystallized from ethanol to give (**5**, **6**) and (**8a**–**c**), respectively.

### Alternative method for synthesis of 3-[(2,4-dimethyl-1,3-thiazol-5-yl)carbonyl]-4,5-dihydroisoxazole-5-carboxamide (**6**)

A mixture of 3-[(2,4-dimethyl-1,3-thiazol-5-yl)carbonyl]-4,5-dihydroisoxazole-5-carbonitrile 5 (0.5 g) and dilute sulfuric acid (5 ml) were stirred at room temperature for 1 h, and then poured onto crushed ice (20 g). The resulting solid was collected and recrystallized from ethanol to give a product identical in all aspects (m.p., mixed m.p., and spectra) with (**6**).

### 3-[(2,4-Dimethyl-1,3-thiazol-5-yl)carbonyl]-4,5-dihydroisoxazole-5-carbonitrile (**5**)

Yellow solid; Yield (73 %); m.p. 101 °C. IR (KBr) ν_max_: 2916, 2846 (CH), 1665 (CO) cm^−1^; ^1^H NMR (DMSO-*d*_6_): 2.57 (s, 3H, CH_3_) 2.80, (s, 3H, CH_3_), 2.90–2.94 (d, 2H, CH_2_, *J* = 10 Hz, isoxazoline C-4) and 3.85 (t, 1H, *J* = 10 Hz, isoxazoline C-5); MS *m/z* (%):234 (M^+^, 70).Anal. Calcd for C_10_ H_9_N_3_O_2_S (235.26): C, 51.05; H, 3.86; N, 17.86; S, 13.63; Found: C, 50.80; H, 3.84; N, 17.89; S, 13.67 %.

### 3-[(2,4-Dimethy-1,3-thiazol-5-yl)carbonyl]-4,5-dihydroisoxazole-5-carboxamide (**6**)

Red solid; yield (66 %); m.p. 168 °C. IR (KBr) ν_max_: 3350, 3170 (NH_2_), 2920, 2856 (CH), 1680 (CO) cm^−1^; ^1^H NMR (DMSO-*d*_6_): 2.46 (s, 3H, CH_3_), 2.79 (s, 3H, CH_3_), 3.54–3.60 (dd, 2H, *J* = 10.98 Hz), 5.20–5.29 (t, 1H, *J* = 8.72 Hz), 5.95 (s, br., 1H), 6.45 (s, br., 1H); MS *m/z* (%): 253 (M^+^, 67).Anal. Calcd for C_10_ H_11_ N_3_ O_3_ S (253.27): C, 47.42; H, 4.38; N, 16.59; S, 12.66; found: C, 47.40; H, 4.36; N, 16.61; S, 12.68 %.

### 3-[(2,4-Dimethyl-1,3-thiazol-5-yl)carbonyl]-5-phenyl-3aH-pyrrolo[3,4-d]isoxazole-4,6(5H,6aH)-dione (**8a**)

Yellow solid; yield (76 %); m.p. 150 °C. IR (KBr) ν_max_: 2926, 2855 (CH), 1717, 1639 (CO’s), 1638 (C=N) cm^−1^; ^1^H NMR (DMSO-d_6_): 2.66 (s, 3H, CH_3_), 2.92 (s, 3H, CH_3_), 5.23–5.24 (d, 1H, J = 7.4 Hz, isoxazoline C-4), 5.81–5.88 (d, 1H, J = 7.4 Hz, isoxazoline C-5), 7.22–7.33 (m, 5H, ArH’s); MS *m/z* (%): 355 (M^+^, 77).Anal. Calcd for C_17_ H_13_ N_3_ O_4_ S (355.36): C, 57.46; H, 3.69; N, 11.82; S, 9.02; found: C, 57.44; H, 3.71; N, 11.84; S, 8.99 %.

### 3-[(2,4-Dimethyl-1,3-thiazol-5-yl)carbonyl]-5-(4-methylphenyl)-3aH-pyrrolo[3,4-d]isoxazole-4,6(5H,6aH)-dione (**8b**)

Yellow solid; yield (80 %); m.p. 156 °C. IR (KBr) ν_max_: 2923, 2853 (CH), 1719, 1633 (CO’s), 1637 (C=N) cm^−1^; ^1^H NMR (DMSO-d_6_): 2.32 (s, 3H, 4-CH_3_C_6_H_4_), 2.54 (s, 3H, CH_3_), 2.89 (s, 3H, CH_3_), 5.18–5.22 (d, 1H, J = 9.78 Hz, isoxazoline C-4), 5.75–5.79 (d, 1H, J = 9.70 Hz, isoxazoline C-5) and 7.18–7.29 (m, 4H, ArH’s); MS *m/z* (%): 369 (M^+^, 90*).*Anal. Calcd for C_18_ H_15_ N_3_ O_4_ S (369.39): C, 58.53; H, 4.09; N, 11.38; S, 8.68; found: C, 58.55; H, 4.12; N, 11.40; S, 8.70 %.

### 3-[(2,4-Dimethyl-1,3-thiazol-5-yl)carbonyl]-5-(4-methoxyphenyl)-3aH-pyrrolo[3,4-d]isoxazole-4,6(5H,6aH)-dione (**8c**)

Yellow solid; yield (77 %); m.p. 165 °C. IR (KBr) ν_max_: 2926, 2918, 2849 (CH), 1716, 1634 (CO’s), 1640 (C=N) cm^−1^; ^1^H NMR (DMSO-d_6_): 2.52 (s, 3H, CH_3_), 2.89 (s, 3H, CH_3_), 3.83 (s, 3H, 4-OCH_3_C_6_H_4_), 5.15–5.17 (d, 1H, J = 7.34 Hz, isoxazoline C-4), 5.73–5.77 (d, 1H, J = 9.40 Hz, isoxazoline C-5) and 6.95–7.29 (m, 4H, ArH’s); MS *m/z* (%): 385 (M^+^, 88).Anal. Calcd for C_18_ H_15_ N_3_ O_5_ S (385.39): C, 56.10; H, 3.92; N, 10.90; S, 8.32; Found: C, 56.12; H, 3.94; N, 10.88; S, 8.34 %.

### Synthesis of sodium salt of 3-hydroxy-1-(2,4-dimethylthiazol-5-yl)prop-2-en-1-one (**10**), [37]

A mixture of 1-(2,4-dimethylthiazol-5-yl)ethanone (**9**) (1.55 g, 10 mmol) and ethylformate (0.74 g, 10 mmol) in dry ether (20 ml) was added portion wise while stirring to solution sodium methoxide (0.54 g, 10 mmol) in dry ether (10 ml) at 0–5 °C. The resulting solid was collected, dried, and was used without purification.

### Synthesis of 3-(dimethylamino)-1-(2,4-dimethyl)(1,3-thiazol-5-yl))prop-2-en-1-one (**11**)

A mixture of 1-(2,4-dimethyl-1,3-thiazol-5-yl)ethanone (**9**) (1.55 g, 0.01 mol) and dimethylformamide-dimethylacetal (1.47 g, 0.01 mol) were refluxed in dry xylene (10 ml) for 4 h. The hot solution was evaporated to its half volume and then cooled. The resulting solid was collected and recrystallized from ethanol to give (**11**).

### 3-(Dimethylamino)-1-(2,4-dimethyl)(1,3-thiazol-5-yl))prop-2-ene-1-one (**11**)

Yellow solid; yield (69 %); m.p. 103 °C. IR (KBr) ν_max_: 2904 (CH) and 1655 (CO conjugated) cm^−1^; ^1^H NMR (DMSO-*d*_6_): 2.49 (s, 3H, CH_3_), 2.78 (s, 3H, CH_3_), 2.98 (s, 3H, = NCH_3_), 3.15 (s, 3H, = NCH_3_), 5.49–5.54 (d, 1H, *J* = 12 Hz, CH=CH–N) and 6.90–7.28 (d, 1H, *J* = 12 Hz, CH=CH–N); MS *m/z* (%): 210 (M^+^, 86).Anal. Calcd for C_10_H_14_N_2_OS (210.29): C, 57.11; H, 6.71; N, 13.32; S, 15.25; found: C, 57.13; H, 6.69; N, 13.34; S, 15.27 %.

### Synthesis of 5-(2,4-dimethyl-1,3-thiazol-5-yl)-2-phenylpyrazolo[1,5-a]pyrimidine** (12a)** and 5-(2,4-dimethyl-1,3-thiazol-5-yl)-3-phenylpyrazolo[1,5-a]pyrimidine (**12b**)

#### Method A

A mixture of the sodium salt (**10**) (1.26 g, 5 mmol) and the appropriate amount of 3-amino-4-phenylpyrazole or 3-amino-5-phenylpyrazole (5 mmol) in a solution of piperidenum acetate [piperidine (2.5 ml)], water (5 ml), and acetic acid (2.5 ml) was heated under reflux for about 10 min. Then acetic acid (1.5 ml) was added while boiling, and the resulting solid was collected and recrystallized from the appropriate solvents to give (**12a**) and (**12b**), respectively.

#### Method B

An equimolar amount of 3-(dimethylamino)-1-(2,4-dimethyl)(1,3-thiazol-5-yl))prop-2-ene-1-one (**11**). (1.05 g, 5 mmol), the appropriate amount of 3-amino-4-phenylpyrazole or 3-amino-5-phenylpyrazole (5 mmol) and ammonium acetate (5 mmol) in acetic acid (10 ml) was heated under reflux for 4 h. The resulting solid was collected and recrystallized from the appropriate solvent to give products identical in all aspects (m.p., mixed m.p., and spectra) with (**12a**) and (**12b**).

### 5-(2,4-Dimethyl-1,3-thiazol-5-yl)-2-phenylpyrazolo[1,5-a]pyrimidine (**12a**)

Yellow solid; yield (79 %); m.p. 107 °C. IR (KBr) ν_max_: 3317 (NH) 3076, 2998 (CH, aromatic and aliphatic), 1628 (CN), 1343 (CH_3_) cm^−1^; ^1^H NMR (DMSO-*d*_6_): 2.59 (s, 3H, CH_3_), 2.83 (s, 3H, CH_3_), 6.53 (s, 1H, pyrazole H-4), 7.13 (d, 1H, *J* = 4 Hz, pyrimidine H-5), 7.54–7.92 (m, 5H, ArH’s) and 8.74 (d, 1H, 8 Hz, pyrimidine H-6); MS *m/z* (%): 306 (M^+^, 48).Anal. Calcd for C_17_H_14_N_4_S (306.38): C, 66.64; H, 4.61; N, 18.29; S, 10.47; found: C, 66.66; H, 4.63; N, 18.31; S, 10.45 %.

### 5-(2,4-Dimethyl-1,3-thiazol-5-yl)-3-phenylpyrazolo[1,5-a]pyrimidine (**12b**)

Red solid; Yield (69 %); m.p. 109 °C. IR (KBr) ν_max_: 3315 (NH), 3057, 2996 (CH, aromatic and aliphatic), 1624 (CN), 1343 (CH_3_) cm^−1^; ^1^H NMR (DMSO-*d*_6_): 2.56 (s, 3H, CH_3_), 2.84 (s, 3H, CH_3_), 7.14 (d, 1H, *J* = 4 Hz, pyrimidine H-5), 7.56–7.78 (m, 5H, ArH’s), 8.74 (d, 1H, *J* = 8 Hz, pyrimidine H-6 and 9.05 (s, 1H, pyrazole H-3); MS *m/z* (%): 306 (M^+^, 44).Anal. Calcd for C_17_H_14_N_4_S (306.38): C, 66.64; H, 4.61; N, 18.29; S, 10.47; found: C, 66.63; H, 4.63; N, 18.27; S, 10.46 %.

### Synthesis of 1-phenyl-4-(2,4-dimethyl)thiazol-5-yl-3-substituted pyrazoles (**20a**–**d**), [38]

Equimolar amounts of each of (**11**) and the appropriate hydrazonoyl halides (**13**–**16**) (0.005 mol) were refluxed in dry toluene containing triethylamine for 3 h. The hot solution was filtered off and the filtrate was evaporated and triturated with petroleum ether (40–60 °C). The resulting solid was collected and crystallized from ethanol to give (**20a**–**d**), respectively.

### Ethyl 4-[(2,4-dimethyl-1,3-thiazol-5-yl)carbonyl]-1-phenyl-1H-pyrazole-3-carboxylate (**20a**)

Yellow solid; yield (73 %); m.p. 140 °C. IR (KBr) ν_max_: 3450 (NH), 3088, 2996 (CH), 1674 (CO) and 1597 (C=C) cm^−1^; ^1^H NMR (DMSO-*d*_6_): 1.22 (t, 3H, CH_3_, *J* = 7 Hz), 2.44 (s, 3H, CH_3_), 2.69 (s, 3H, CH_3_), 4.33 (q, 2H, CH_2_, *J* = 7 Hz), 7.44–7.89 (m, 5H, ArH’s) and 8.19 (s, 1H, pyrazole C-5); MS *m/z* (%): 355 (M^+^, 56).Anal. Calcd for C_18_H_17_N_3_O_3_S (355.41): C, 60.83; H, 4.82; N, 11.82; S, 9.02; found: C, 60.85; H, 4.80; N, 11.84; S, 9.15 %.

### 1-{4-[(2,4-Dimethyl-1,3-thiazol-5-yl)carbonyl]-1-phenyl-1H-pyrazol-3-yl}ethanone (**20b**)

Yellow solid; yield (70 %); m.p. 119 °C. IR (KBr) ν_max_: 3039, 2985 (CH), 1648 (CO conjugated) and 1599 (C=C) cm^−1^; ^1^H NMR (DMSO-*d*_6_): 2.57 (s, 3H, CH_3_), 2.69 (s, 3H, CH_3_), 2.78 (s, 3H, CH_3_), 7.15–7.75 (m, 5H, ArH’s) and 8.21 (s, 1H, pyrazole C-5); MS *m/z* (%): 325 (M^+^, 60).Anal. Calcd for C_17_H_15_N_3_O_2_S (325.38): C, 62.75; H, 4.65; N, 12.91; S, 9.85; found: C, 62.77; H, 4.66; N, 12.89; S, 9.87 %.

### (2,4-Dimethyl-1,3-thiazol-5-yl)[1-phenyl-3-(phenylcarbonyl)-1H-pyrazol-4-yl]methanone (**20c**)

Yellow solid; Yield (78 %); m.p. 157 °C. IR (KBr) ν_max_: 3058, 2919 (CH), 1645 (CO conjugated) and 1598 (C=C) cm^−1^; ^1^H NMR (DMSO-*d*_6_): 2.44 (s, 3H, CH_3_), 2.7 (s, 3H, CH_3_), 7.21–8.11 (m, 10H, ArH’s) and 8.31 (s, 1H, pyrazole C-5); MS *m/z* (%): 387 (M^+^, 80). Anal. Calcd for C_22_ H_17_N_3_O_2_S (387.45): C, 68.20; H, 4.42; N, 10.85; S, 8.28; found: C, 68.22; H, 4.40; N, 10.87; S, 8.30 %.

### 4-[(2,4-Dimethyl-1,3-thiazol-5-yl)carbonyl]-N,1-diphenyl-1H-pyrazole-3-carboxamide (**20d**)

Pale yellow solid; yield (79 %); m.p. 190 °C. IR (KBr) ν_max_: 3438 (NH), 3065, 2993 (CH), 1677 (CO) and 1595 (C=C) cm^−1^; ^1^H NMR (DMSO-*d*_6_): 2.48 (s, 3H, CH_3_), 2.79 (s, 3H, CH_3_), 7.13–7.87 (m, 10H, ArH’s), 8.35 (s, 1H, pyrazole C-5) and 10.71 (s, 1H, NH); MS *m/z* (%): 402 (M^+^, 73).Anal. Calcd for C_22_H_18_N_4_O_2_S (402.46): C, 65.65; H, 4.51; N, 13.92; S, 7.97; found: C, 65.67; H, 4.49; N, 13.90; S, 7.99 %.

### Synthesis of pyrazolo[3,4-d]pyridazines (**22a**–**c**), [38]

An appropriate amount of substituted pyrazole (**20a**-**d**). (0.5 g) and hydrazine hydrate (1 ml) in ethanol (15 ml) was refluxed for 1 h. The resulting solid was collected and recrystallized from ethanol to give the pyrazolo[3,4-d]pyridazines (**22a**-**c**).

### 4-(2,4-Dimethyl-1,3-thiazol-5-yl)-2-phenyl-2H-pyrazolo[3,4-d]pyridazin-7-ol (**22a**)

Yellow solid; yield (78 %); m.p. 243 °C. IR (KBr) ν_max_: 3450 (NH), 3088, 2996 (CH), 1674 (CO) and 1597 (C=C) cm^−1^; ^1^H NMR (DMSO-d_6_): 2.45 (s, 3H, CH_3_), 2.72 (s, 3H, CH_3_), 7.22–8.12 (m, 5H, ArH’s), 8.54 (s, 1H, pyrazole C-5) and 10.11 (s, 1H, NH); MS *m/z* (%): 323 (M^+^, 50).Anal. Calcd for C_16_H_13_N_5_OS (323.37): C, 59.43; H, 4.05; N, 21.66; S, 9.92; found: C, 59.45; H, 4.15; N, 21.64; S, 9.90 %.

### 4-(2,4-Dimethyl-1,3-thiazol-5-yl)-7-methyl-2-phenyl-2H-pyrazolo[3,4-d]pyridazine (**22b**)

Yellow solid; yield (75 %); m.p. 190 °C. IR (KBr) ν_max_: 3045, 2991 (CH) and 1587 (C=C) cm^−1^; ^1^H NMR (DMSO-*d*_6_): 2.46 (s, 3H, CH_3_), 2.77 (s, 3H, CH_3_), 3.01 (s, 3H, CH_3_), 7.32–8.11 (m, 5 H, ArH’s) and 8.76 (s, 1H, pyrazole C-5); MS *m/z* (%): 321 (M^+^, 55). Anal. Calcd for C_17_H_15_N_5_S (321.39): C, 63.53; H, 4.70; N, 21.79; S, 9.98; found: C, 63.55; H, 4.72; N, 21.81; S, 10.00 %.

### 4-(2,4-Dimethyl-1,3-thiazol-5-yl)-2,7-diphenyl-2H-pyrazolo[3,4-d]pyridazine (**22c**)

Yellow solid; yield (83 %); m.p. 204 °C. IR (KBr) ν_max_: 3055, 2958 (CH) and 1594 (C=C) cm^−1^; ^1^H NMR (DMSO-d_6_): 2.44 (s, 3H, CH_3_), 2.89 (s, 3H, CH_3_), 7.23–8.75 (m, 10 H, ArH’s) and 8.81(s, 1H, pyrazole C-5); MS *m/z* (%): 383 (M^+^, 63). Anal. Calcd for C_22_ H_17_N_5_S (383.46): C, 68.91; H, 4.47; N, 18.26; S, 8.36; found: C, 68.89; H, 4.49; N, 18.28; S, 8.38 %.

### Antimicrobial activity

The synthesized compounds were tested for their antimicrobial activity against gram positive and gram negative bacteria as well as some fungal-plants. The sensitivity of the selected microorganisms towards the compounds under investigation was determined in vitro culture dissolved in chloroform, Appling the filter paper and hole plate method [39]. The sterile filter paper disc was saturated with 10 μL of 0.5 mg ml^−1^ w/v solution of the compound under investigation in DMF. A comparative study of the biological activity of these compounds with Ampicillin and tetracycline is compiled in Table 3. Generally, all synthesized compounds showed an adequate inhabitory efficiency of growth of gram positive and gram negative bacteria.

**Table 3:** Response of various microorganisms to some synthesized compounds in vitro (culture)

Microorganism/compound no.	*Staphylococcus albus* (G^+^)	*Streptococcus faecalis* (G^+^)	*Bacillus subtilis* (G^+^)	*Echerichia coli* (G^−^)	*Aspergills flvus* (fungus)	*Candida albicans* (fungus)
Ampicillin/tetracycline	34R/27	37/31	33/30	39/34	0.0/0.0	20/37
**3**	16	13	18	18	0.0	16
**5**	19	16	12	17	0.0	13
**8a**	16	15	12	17	0.0	14
**8b**	15	16	14	16	0.0	14
**8c**	17	15	12	17	0.0	13
**12a**	15	14	13	16	0.0	13
**12b**	14	16	13	13	0.0	14
**22a**	16	15	14	15	0.0	14
**22b**	15	18	12	15	0.0	14
**22c**	16	15	14	16	0.0	14

St. reference standard; ampicillin and tetracycline were used as a slandered antibacterial agent and antifungal agent. Values show zone of inhibition in mm. Diameter of the inhibition zones were: high (11–15 mm), moderate (6–10 mm), slight (1–5 mm) and negative (0)

## Abbreviations

m.p.: melting point
CNS: the central nervous system
13DC: 1,3-dipolar cycloaddition
ZPE: the zero-point energy
G: free energies
H: enthalpies
MW: molecular weight
TLC: thin layer chromatography
